# Microbial community dynamics during alfalfa silage with or without clostridial fermentation

**DOI:** 10.1038/s41598-020-74958-1

**Published:** 2020-10-20

**Authors:** Rongrong Li, Di Jiang, Mingli Zheng, Pengjiao Tian, Menghu Zheng, Chuncheng Xu

**Affiliations:** 1 College of Engineering, China Agricultural University, Beijing, 100083 China; 2 Beijing Research and Development Center for Grass and Environment, Beijing Academy of Agriculture and Forestry Sciences, Beijing, 100097 China

**Keywords:** Biotechnology, Microbiology

## Abstract

This study was conducted to examine the effects of *Lactobacillus plantarum* (LP) and sucrose (S) on clostridial community dynamics and correlation between clostridia and other bacteria in alfalfa silage during ensiling. Fresh alfalfa was directly ensiled without (CK) or with additives (LP, S, LP + S) for 7, 14, 28 and 56 days. Clostridial and bacterial communities were evaluated by next-generation sequencing. Severe clostridial fermentation occurred in CK, as evidenced by the high contents of butyric acid, ammonia nitrogen, and clostridia counts, whereas all additives, particularly LP + S, decreased silage pH and restrained clostridial fermentation. *Clostridium perfringens* and *Clostridium butyricum* might act as the main initiators of clostridial fermentation, with *Clostridium tyrobutyricum* functioning as the promoters of fermentation until the end of ensiling. *Clostridium tyrobutyricum* (33.5 to 98.0%) dominated the clostridial community in CK from 14 to 56 days, whereas it was below 17.7% in LP + S. *Clostridium* was negatively correlated with the genus *Lactobacillus*, but positively correlated with the genera *Enterococcus, Lactococcus* and *Leuconostoc*. Insufficient acidification promoted the vigorous growth of *C. tyrobutyricum* of silage in later stages, which was mainly responsible for the clostridial fermentation of alfalfa silage.

## Introduction

Ensiling is a traditional method of fresh forage preservation based on anaerobic fermentation by lactic acid bacteria (LAB). After sealing, trapped oxygen is consumed through plant and aerobic microbial respiration, creating an anaerobic environment that terminates respiration; clostridia and enterobacteria, which can grow under anaerobic conditions, compete for available nutrients with LAB, particularly in high moisture forages^1^. If initial acidification caused by lactic acid fermentation fails to effectively prevent clostridial proliferation in silage, clostridial fermentation occurs, resulting in poorly preserved silage. Clostridial fermentation may cause anaerobic deterioration of silage either through undesirable rancid odor by butyric producing clostridia such as *Clostridium butyricum*, *Clostridium beijerinckii*, and *Clostridium tyrobutyricum* or through accumulation of ammonia and amines in clostridia such as *Clostridium perfringens* and *Clostridium sporogenes*^2,3^. Moreover, the potential risks to animal health and milk contamination mainly caused by *Clostridium botulinum* and *C. tyrobutyricum* can result in great economic losses to the dairy industry when clostridial silage is fed^4,5^.

Silage quality is highly dependent on the microbial community and its metabolites present during the ensiling process^6^. A better understanding of the microbial community may lead to the development of strategies for enhancing silage preservation and reducing clostridial risk. In previous studies, species such as *C. butyricum*, *C. tyrobutyricum*, *C. perfringens*, and *C. sporogenes* were isolated and identified from a variety of silage samples using conventional culture-based methods^2,7,8^. However, this method typically underestimates the level of clostridial diversity because of most naturally present species cannot be cultivated. In the past decade, developments in molecular technology have allowed researchers to understand the complete structure of the clostridial community present in silage. Rossi et al.^9^ revealed that *C*. *perfringens* was strongly associated with the poor fermentation quality of Verona (Italian) farm-made alfalfa silage based on random amplified polymorphic DNA polymerase chain reaction techniques. Zheng et al.^10^ found that *C. perfringens* and *C. sporogenes* mainly contributed to the clostridial fermentation of alfalfa silage according to denaturing gradient gel electrophoresis analysis. Although these investigations have expanded our knowledge of clostridial populations, the information obtained was limited to the species abundance associated with the complex clostridial community^11^. Next-generation sequencing (NGS) has been widely used to examine the occurrence and abundance of whole microbial communities in silage. Recent studies applied NGS to examine the bacterial community of moist forage silage^12,13^; however, clostridial diversity was not evaluated in these studies as their relative abundance is often far less than 1% in complex microbial ecosystems. The presence of specific-clostridia primers seems to create the conditions for comprehensive understanding of clostridial diversity in silage^14^.

*Lactobacillus plantarum* (LP) and sucrose (S) are widely used to promote lactic acid fermentation and restrict clostridial fermentation, which improvs the silage quality of moist forage crops^15,16^. Most previous studies only reported how changes in the bacterial community affect the quality of silage^6,17,18^, whereas the effects of these additives on clostridial diversity are unclear. We previously analyzed the clostridial community dynamics of alfalfa silage following addition of both LP and S. However, the clostridial community dynamics were not detected when LP or S was added separately. Furthermore, limited information is available regarding the correlation between clostridia and other bacteria during alfalfa silage. In this study, we comprehensively analyzed the clostridial community dynamics by NGS method and the correlation between clostridia and other bacteria in ensiled alfalfa containing LP and S for modulation.

## Results

### Chemical and microbial composition of pre-ensiled alfalfa

The dry matter (DM) and water-soluble carbohydrate (WSC) contents in fresh alfalfa were 224 g/kg fresh weight (FM) and 47.2 g/kg DM (Table 1). Crude protein, neutral detergent fiber and acid detergent fiber contents were 210, 387 and 265 g/kg DM, respectively. The numbers of epiphytic LAB and clostridia in fresh alfalfa were 4.66 and 1.30 log_10_ cfu/g FM, respectively.

**Table 1:** Chemical and microbial composition of pre-ensiled alfalfa.

Items	Alfalfa
**Chemical composition (g/kg DM)**	
Dry matter (g/kg FM)	224 ± 1.53
Crude protein	210 ± 2.64
Water soluble carbohydrates	47.2 ± 0.19
Neutral detergent fiber	387 ± 8.34
Acid detergent fiber	265 ± 4.57
**Microbial composition (log**_**10**_** cfu/g FM)**	
Lactic acid bacteria	4.66 ± 0.36
Clostridia	1.30 ± 0.11
Enterobacteria	5.11 ± 0.38

Means ± standard deviation, n = 3; DM, dry matter; FM, fresh matter; cfu, colony forming units.

### Fermentation quality and microbial analysis of alfalfa silages

All treatments had higher lactic acid content and lactic to acetic ratio (*P* < 0.05) and LP + S had the highest lactic acid content (*P* < 0.05) (Table 2). The S and LP + S groups showed lower silage pH and ammonia nitrogen (NH_3_–N) content compared to CK and LP, particularly CK (*P* < 0.05). After 7 days of ensiling, butyric acid content was highest in CK, followed by LP, but was undetectable in S and LP + S (*P* < 0.05). As the fermentation continued, butyric acid and NH_3_–N contents significantly increased in CK (*P* < 0.05), with value of 6.67 g/kg DM and 131 g/kg total nitrogen (TN) at 56 days. In S and LP + S, however, NH_3_–N content was no more than 23.1 g/kg TN during ensiling. The lactic acid content continuously increased in CK from 0 to 28 days, and then showed a decrease at 56 days; the opposite trend was observed for silage pH during ensiling.

**Table 2:** Effect of different additives on fermentation quality (g/kg DM) of alfalfa silage during ensiling.

Items	Treatment	Storage period				SEM	*P*-value		
		7	14	28	56		T	S	T × S
pH	CK	5.54^aA^	5.30^aB^	5.21^aC^	5.28^aB^	0.051	< 0.001	< 0.001	0.003
	LP	5.10^bA^	4.99^bB^	4.85^bC^	4.80^bC^				
	S	4.34^cA^	4.27^cB^	4.19^cC^	4.07^cD^				
	LP + S	4.20^dA^	4.07^ dB^	4.03^dBC^	3.98^dC^				
Lactic acid	CK	13.7^dC^	19.4^ dB^	23.6^dA^	13.4^dC^	0.337	< 0.001	< 0.001	< 0.001
	LP	21.9^cC^	27.4^cB^	30.8^cA^	31.1^cA^				
	S	45.9^bD^	51.5^bC^	60.5^bB^	71.3^bA^				
	LP + S	65.8^aB^	78.3^aA^	79.1^aA^	80.2^aA^				
Acetic acid	CK	16.6^aC^	22.9^aB^	24.2^aB^	30.6^aA^	0.078	0.003	< 0.001	< 0.001
	LP	12.3^bAB^	12.0^cB^	13.2^cAB^	14.5^cA^				
	S	16.2^aC^	18.0^bBC^	18.9^bB^	21.1^bA^				
	LP + S	11.2^bB^	12.1^cA^	12.5^cA^	12.6^cA^				
LA/AA	CK	0.83^dA^	0.85^cA^	0.98^dA^	0.44^ dB^	0.209	< 0.001	0.005	0.010
	LP	1.78^cC^	2.28^bAB^	2.33^cA^	2.14^cB^				
	S	2.83^bB^	2.86^bB^	3.25^bAB^	3.38^bA^				
	LP + S	5.88^aB^	6.47^aA^	6.32^aA^	6.37^aA^				
PA	CK	0.75^aD^	0.98^aC^	1.35^aB^	1.86^aA^	0.065	< 0.001	< 0.001	< 0.001
	LP	0.07^cD^	0.25^cC^	0.43^cB^	0.73^bA^				
	S	0.41^bB^	0.78^bA^	0.76^bA^	0.87^bA^				
	LP + S	0.01^cC^	0.12^cC^	0.31^cB^	0.49^cA^				
Butyric acid	CK	1.86^aD^	3.05^aC^	4.10^aB^	6.67^aA^	0.097	< 0.001	0.006	0.012
	LP	0.00^bB^	0.12^bB^	0.63^bA^	0.85^bA^				
	S	0.00^b^	0.00^c^	0.00^c^	0.00^c^				
	LP + S	0.00^b^	0.00^c^	0.00^c^	0.00^c^				
NH_3_–N (g/kg TN)	CK	87.2^aD^	95.2^aC^	108^aB^	131^aA^	0.537	< 0.001	< 0.001	< 0.001
	LP	36.1^bD^	54.3^bC^	65.7^bB^	78.3^bA^				
	S	11.7^cC^	16.2^cB^	19.1^cB^	23.1^cA^				
	LP + S	8.26^dC^	12.8^ dB^	15.0^dAB^	17.2^dA^				

^a-d^Means within a column with different superscripts differ (*P* < 0.05); ^A-E^ Means within a raw with different superscripts differ (*P* < 0.05). LP, *Lactobacillus plantarum*; S, sucrose; LP + S, the addition of both sucrose and *Lactobacillus plantarum*; TN, total nitrogen; DM, dry matter; FM, fresh matter; LA/AA: lactic to acetic ratio; PA, propionic acid; T: effect of treatment; S: effect of storage period; T × S: interaction between treatment and storage period; SEM, standard error of means.

The DM content was higher in the LP + S samples than in the CK and LP samples over the whole ensiling period (*P* < 0.05), whereas no significant difference in DM was observed between the S and LP + S samples (*P* > 0.05) (Table 3). A higher content of WSC was observed in S than in LP during the first 14 days of ensiling (*P* < 0.05), but did not differ from that of LP + S (*P* > 0.05). *Clostridia* and enterobacteria counts were higher in LP and CK than in S and LP + S (*P* < 0.05), but were undetectable in LP + S over the whole ensiling period (*P* < 0.05). Compared with LP and CK, LP + S showed a higher LAB count by 7 days, with the opposite tendency detected after 14 days of ensiling (*P* < 0.05).

**Table 3:** Effect of different additives on dry matter (g/kg FM), WSC (g/kg DM) and microbial counts (log_10_ cfu/g FM) of alfalfa silage during ensiling.

Items	Treatment	Storage period				SEM	*P*-value		
		7	14	28	56		T	S	T × S
Dry matter	CK	211^cA^	209^cAB^	207^cAB^	201^cB^	0.109	0.004	0.043	0.335
	LP	221^b^	218^b^	217^b^	214^b^				
	S	228^ab^	225^ab^	227^a^	226^a^				
	LP + S	230^a^	229^a^	228^a^	229^a^				
WSC	CK	16.1^bcA^	14.5^abA^	12.3^AB^	9.73^B^	0.051	0.022	0.009	0.054
	LP	13.1^cA^	12.4^bAB^	11.4^AB^	8.83^B^				
	S	21.3^aA^	17.2^aB^	13.6^BC^	11.2^C^				
	LP + S	17.5^abA^	14.4^abAB^	11.1^BC^	8.80^C^				
LAB	CK	8.29^cA^	8.16^AB^	8.09^abAB^	7.90^aB^	0.195	0.003	0.006	0.019
	LP	8.63^bA^	8.23^B^	8.11^aBC^	7.89^aC^				
	S	8.11^cA^	8.02^AB^	7.72^bcB^	7.55^bB^				
	LP + S	8.94^aA^	8.06^B^	7.59^cC^	7.36^bC^				
Clostridia	CK	2.13^aD^	2.57^aC^	3.08^aB^	3.64^aA^	0.019	0.016	0.020	0.038
	LP	1.72^bD^	2.11^bC^	2.45^bB^	2.80^bA^				
	S	1.38^c^	1.30^c^	< 1.30	< 1.30				
	LP + S	< 1.30	< 1.30	< 1.30	< 1.30				
Enterobacteria	CK	7.22^aA^	5.69^aB^	4.91^aC^	4.17^aD^	0.033	0.011	0.009	0.022
	LP	5.03^bA^	4.37^bB^	3.64^bC^	3.05^bD^				
	S	< 2.40	< 2.40	< 2.40	< 2.40				
	LP + S	< 2.40	< 2.40	< 2.40	< 2.40				

^a–d^ Means within a column with different superscripts differ (*P* < 0.05); ^A–E^Means within a raw with different superscripts differ (*P* < 0.05); LAB, lactic acid bacteria; LP, *Lactobacillus plantarum*; S, sucrose; LP + S, the addition of both sucrose and *Lactobacillus plantarum*; WSC, water soluble carbohydrates; DM, dry matter; FM, fresh matter; T: effect of treatment; S: effect of storage period; T × S: interaction between treatment and storage period; SEM, standard error of means.

### Bacterial diversity dynamics of alfalfa silage

**Figure 1:**
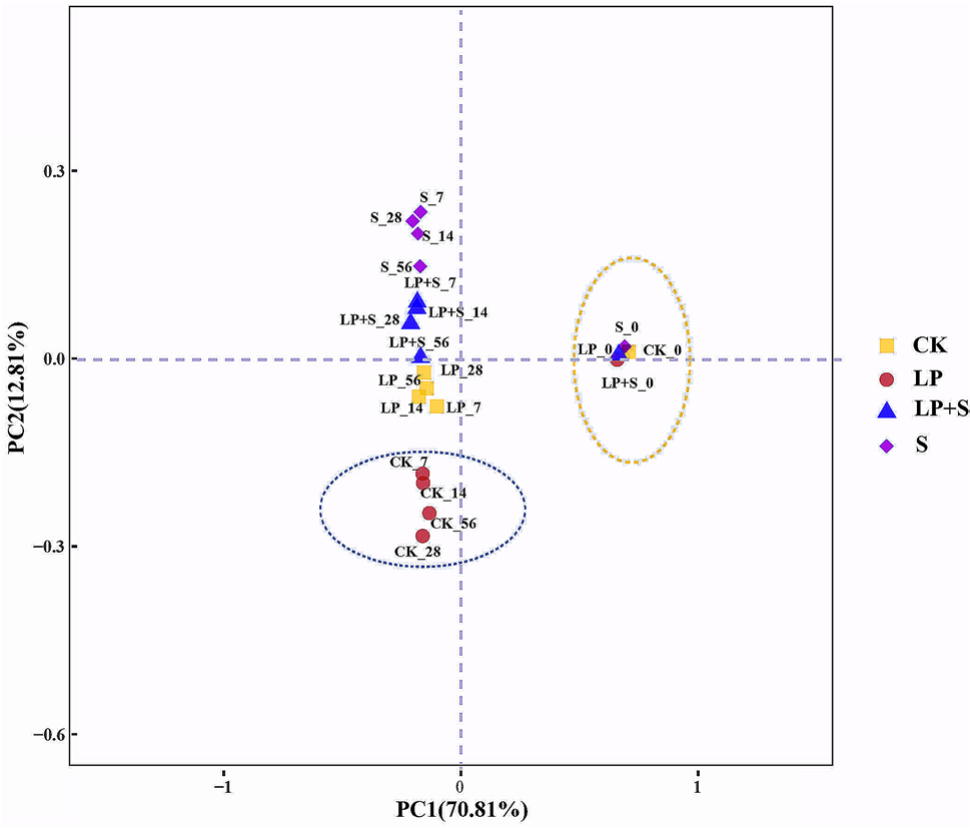
The principal component analysis of the bacterial community diversity at the genus level. CK, control; LP, *Lactobacillus plantarum*; S, sucrose; LP + S, the addition of both sucrose and *Lactobacillus plantarum*.

A total of 2,539,941 quality sequence reads were observed by high-throughput amplicon sequencing of the 16S rRNA gene (V3–V4 regions) in all samples. These reads were clustered into a total of 3952 operational taxonomic units (OTUs) at 97% sequence similarity (Table 4). The average Good’s coverage for all samples was around 1.0, indicating that the sampling depth adequately captured most of the bacterial communities. The OTUs, as an index assessing the richness of bacterial communities, decreased in alfalfa silage treated with LP + S at 7 and 56 days compared with that in CK (*P* < 0.05). The Chao1 and Shannon indices in S and CK at 56 days were significantly higher than those in LP + S (*P* < 0.05). Distinct separation was observed between the raw materials and treated silages (Fig. 1). Among the silage samples, there was a clear separation between CK and other treated silages.

**Table 4:** Alpha diversity of bacterial community in alfalfa silage during ensiling.

Treatments	Days	Reads	OTUs	Shannon	Chao	Coverage
CK	0	43223^bB^	273^a^	3.68^A^	353^ab^	1.00
	7	44199^a^^bAB^	207^bA^	3.78^A^	304^bcA^	1.00
	14	49967^aA^	178^b^	3.66	273^cA^	1.00
	28	45102^a^^bA^	205^b^	3.84	364^a^	1.00
	56	41818^bB^	242^abA^	3.99^A^	297^bcA^	1.00
LP	0	49228^aAB^	239^a^	2.72^bB^	345^aA^	0.99
	7	49227^aA^	169^bAB^	3.35^aAB^	247^bAB^	1.00
	14	41338^bB^	165^b^	3.54^a^	263^bAB^	1.00
	28	38220^bB^	195^ab^	3.65^a^	306^ab^	1.00
	56	41787^bB^	176^abAB^	3.46^aAB^	287^abA^	1.00
S	0	45544^aAB^	276^a^	3.38^A^	351^a^	0.99
	7	41118^a^^bB^	184^bAB^	3.37^AB^	289^bA^	1.00
	14	38416^bB^	186^b^	3.62	269^bAB^	1.00
	28	29425^cC^	202^b^	3.75	308^ab^	1.00
	56	39168^bB^	200^bAB^	3.70^aA^	294^abA^	1.00
LP + S	0	50572^aA^	236^a^	2.76^bB^	346^a^	0.99
	7	39182^bB^	136^bB^	2.89^a^^bB^	217^bB^	1.00
	14	37483^bcB^	148^b^	3.32^ab^	210^bB^	1.00
	28	32648^cC^	190^ab^	3.41^a^	301^a^	0.99
	56	48982^aA^	145^bB^	3.04^abB^	227^bB^	1.00
*P*-value	T	0.013	0.035	0.020	0.025	0.487
	S	0.016	0.009	0.031	0.011	0.577
	T × S	0.024	0.083	0.039	0.018	0.578
SEM		745	13	0.044	16	0.0005

^a–d^Means within a column with different superscripts differ (*P* < 0.05); ^A–E^Means within a raw with different superscripts differ (*P* < 0.05). CK, control; LP, *Lactobacillus plantarum*; S, sucrose; LP + S, the addition of both sucrose and *Lactobacillus plantarum*; T: effect of treatment; S: effect of storage period; T × S: interaction between treatment and storage period; SEM, standard error of means.

**Figure 2:**
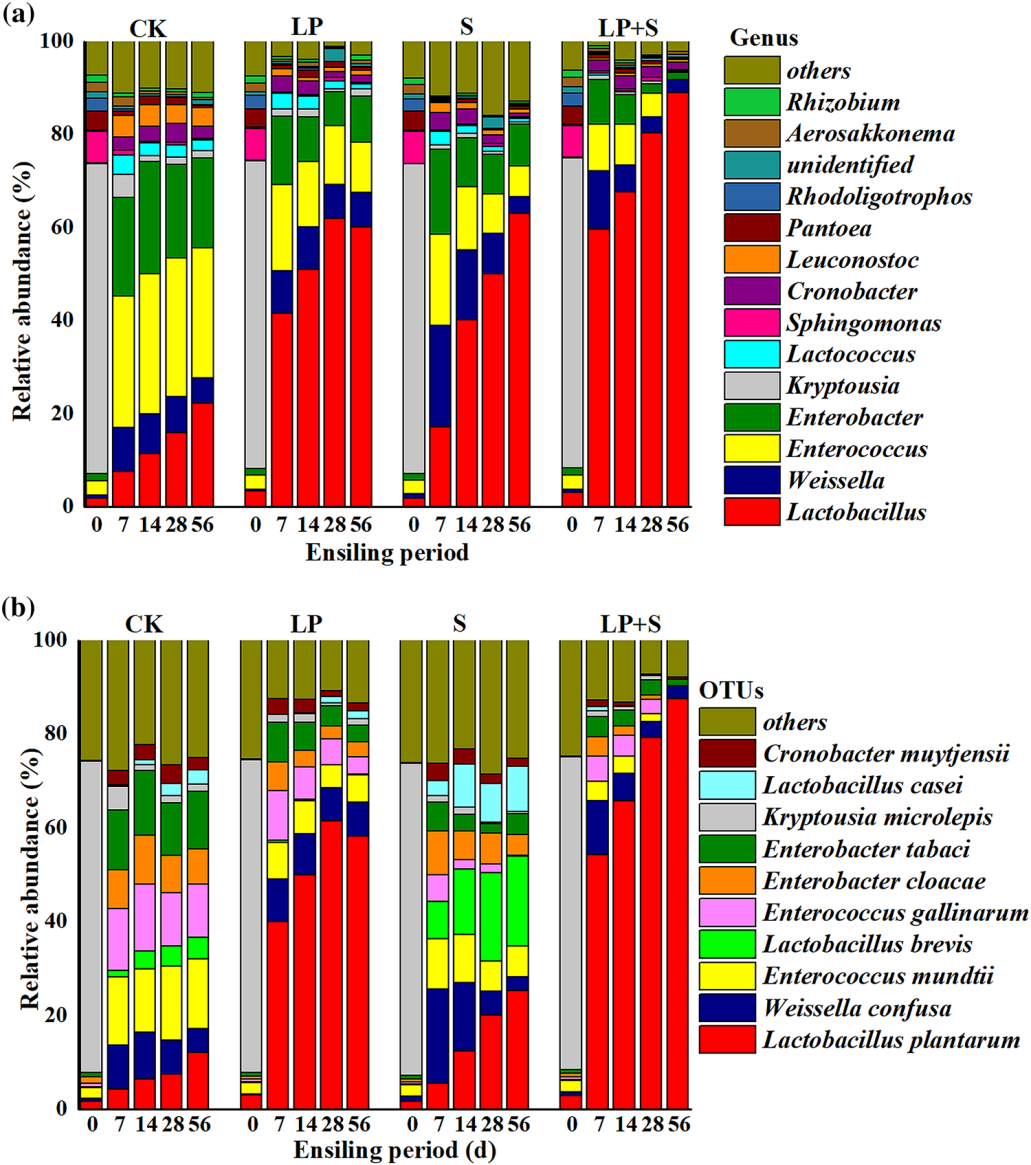
Relative abundance of the bacterial community at the genus (**a**) and species levels (**b**). CK, control; LP, *Lactobacillus plantarum*; S, sucrose; LP + S, the addition of both sucrose and *Lactobacillus plantarum*.

At the genus level, *Kryptousia* (66.7%) and *Sphingomonas* (6.77%) were the main epiphytic bacteria in fresh alfalfa, whereas *Clostridium* accounted for only 0.16% of the total population (Fig. 2a). In CK silages, *Enterococcus* (28.3%) was the most prevalent genus at 7 days, thereafter maintained the most prevalent genera by 56 days; whereas the relative abundance *Lactobacillus* constantly increased as fermentation continued, accounting for 22.3% of the total sequences at 56 days. *Weissella* (21.8%), *Enterococcus* (19.6%), and *Enterobacter* (18.3%) peaked at 7 days in S, and thereafter declined by 56 days; in contrast, *Lactobacillus* increased continuously during the ensiling process. After 28 days of ensiling, *Lactobacillus* peaked (62.1%) in the LP, and then declined to 60.1% at 56 days.

At the species level, *Enterococcus mundtii* (14.7%) and *Enterbacter tabaci* (12.8%) predominated at 7 days and remained relatively constant by 56 days in CK, whereas *L. plantarum* continuously increased over the ensiling period, accounting for 12.2% of the total population at 56 days (Fig. 2b). In LP and LP + S silages, *L. plantarum* increased in relative abundance after ensiling, peaking at 28 and 56 days, respectively. Compared with LP and LP + S, S showed a lower relative abundance of *L. plantarum* over the ensiling period, with a higher relative abundance of *L. brevis* and *L. casei*.

**Figure 3:**
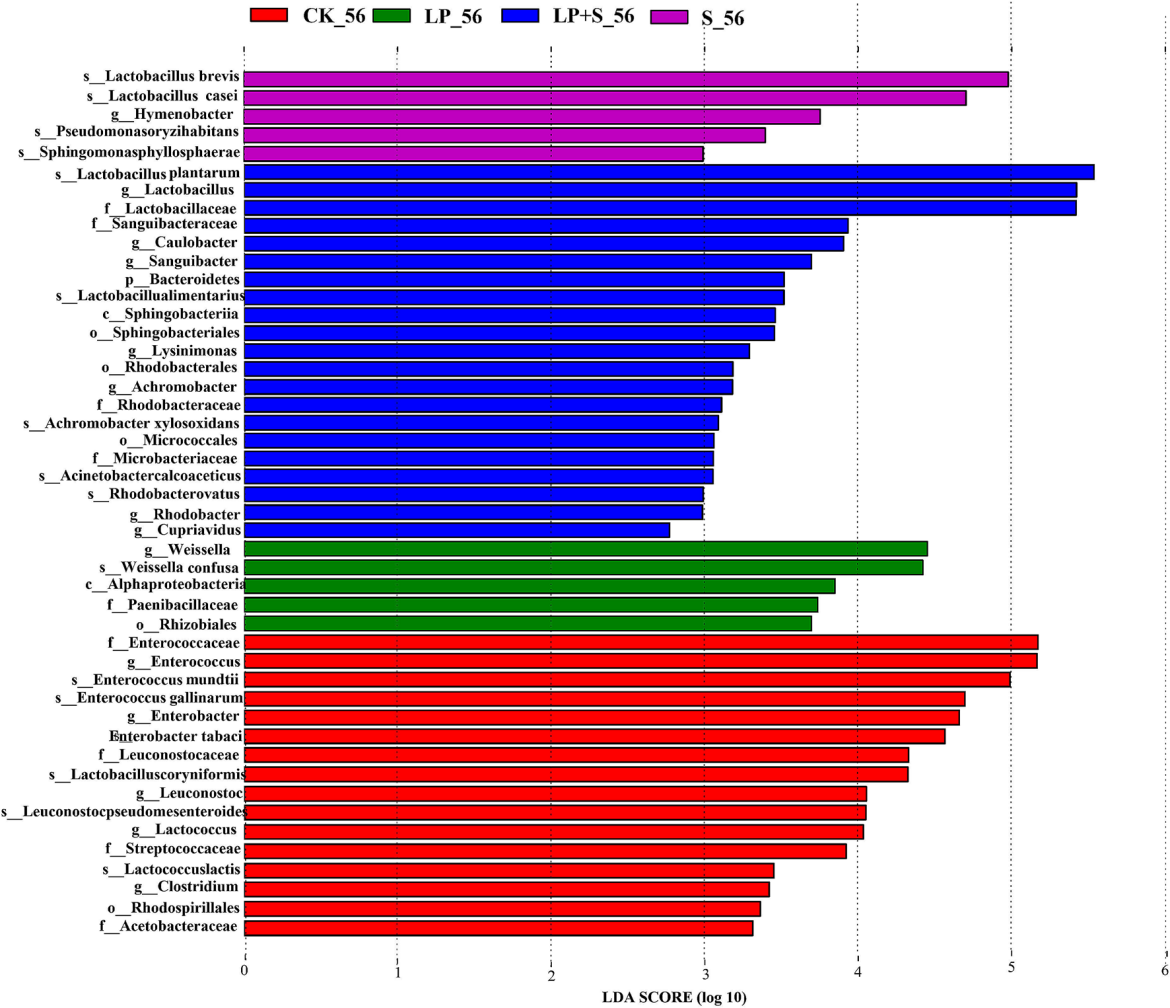
Comparison of bacterial variations using the LEfSe online tool for alfalfa silage at 56 days (CK, control; LP, *Lactobacillus plantarum*; S, sucrose; LP + S, the addition of both sucrose and *Lactobacillus plantarum*).

Differences in bacterial flora among the groups were analyzed by the linear discriminant analysis effect size (LEfSe) method, which was used to determine the bacteria most likely to illustrate the differences among the different treatments in alfalfa silage at 56 days (Fig. 3). *Enterococcus mundtii* was higher in CK; *Weissella confusa* was higher in LP; *L. brevis* was higher in S, and *L. plantarum* was higher in LP + S.

### Clostridial diversity dynamics of alfalfa silage

**Figure 4:**
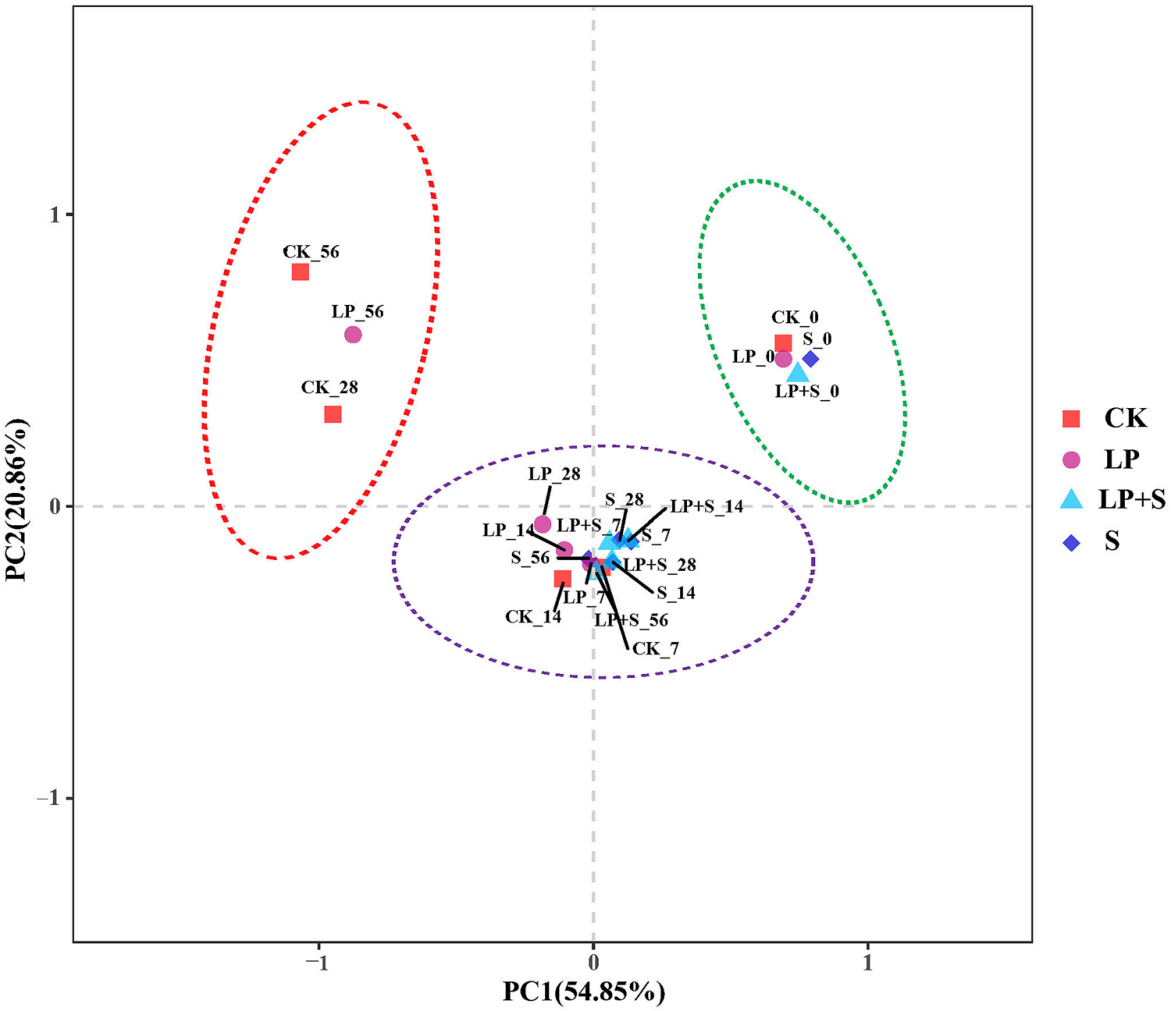
The principal component analysis of the clostridial community diversity at the genus level. CK, control; LP, *Lactobacillus plantarum*; S, sucrose; LP + S, the addition of both sucrose and *Lactobacillus plantarum*.

A total of 445,596 quality sequence reads were observed by high-throughput amplicon sequencing of the 16S rRNA gene (V4–V5 regions) in all samples and these reads were clustered into a total of 420 OTUs at 97% sequence similarity (Table 5). Most of the clostridial community was observed, as indicated by the Good’s coverage values of all samples which were around 1.00. The Chao 1 and Shannon index of the clostridial community in S and LP + S were greater than those in CK after 14 days of ensiling (*P* < 0.05). The treated silage showed lower OUTs at 56 days compared to those in CK (*P* < 0.05). All samples were divided into three groups: the four fresh alfalfa raw materials in the first group, samples of CK at 28 and 56 days and S at 56 days in the second group, with the remaining samples in the last group (Fig. 4).

**Table 5:** Alpha diversity of clostridial community in alfalfa silage during ensiling.

Treatments	Days	Reads	OTUs	Shannon	Chao	Coverage
CK	0	1279^d^	24^ab^	2.93^a^^b^	31^a^	0.99
	7	7044^cA^	26^a^	3.40^a^^A^	33^a^	0.99
	14	4035^ cd^	23^ab^	2.83^bB^	25^abc^	0.99
	28	21154^bA^	18^b^	1.73^cB^	19^b^^B^	1.00
	56	41069^aA^	5^cC^	0.09^dC^	7^cC^	0.99
LP	0	1266^c^	26^a^	3.04^a^	30^a^	0.99
	7	3485^cAB^	23^a^	2.61^a^^bB^	26^a^	1.00
	14	4010^b^^c^	20^ab^	3.08^aAB^	22^ab^	0.99
	28	7998^bB^	19^ab^	1.97^bB^	21^abAB^	0.99
	56	28024^aB^	13^bB^	0.81^cB^	16^bB^	0.99
S	0	1187	25	2.96	32	0.99
	7	4575^AB^	22	2.92^B^	29	0.99
	14	2581	20	3.19^AB^	24	1.00
	28	4099^BC^	22	3.04^A^	29^A^	0.99
	56	3869^C^	19^AB^	3.22^A^	26^A^	0.99
LP + S	0	1243	24	2.97	32	0.99
	7	2065^B^	23	2.94^B^	31	1.00
	14	3855	22	3.23^A^	23	0.99
	28	3394^C^	22	3.01^A^	26^A^	0.99
	56	2300^C^	24^A^	3.17^A^	24^AB^	0.99
*P*-value	T	0.030	0.035	0.027	0.029	0.162
	S	0.019	0.047	0.006	0.037	0.540
	T × S	0.051	0.111	0.102	0.035	0.771
SEM		669	0.911	0.716	1.13	0.0003

^a–d^Means within a column with different superscripts differ (*P* < 0.05); ^A–E^Means within a raw with different superscripts differ (*P* < 0.05). CK, control; LP, *Lactobacillus plantarum*; S, sucrose; LP + S, the addition of both sucrose and *Lactobacillus plantarum*; T, effect of treatment; S, effect of storage period; T × S: interaction between treatment and storage period; SEM, standard error of means.

**Figure 5:**
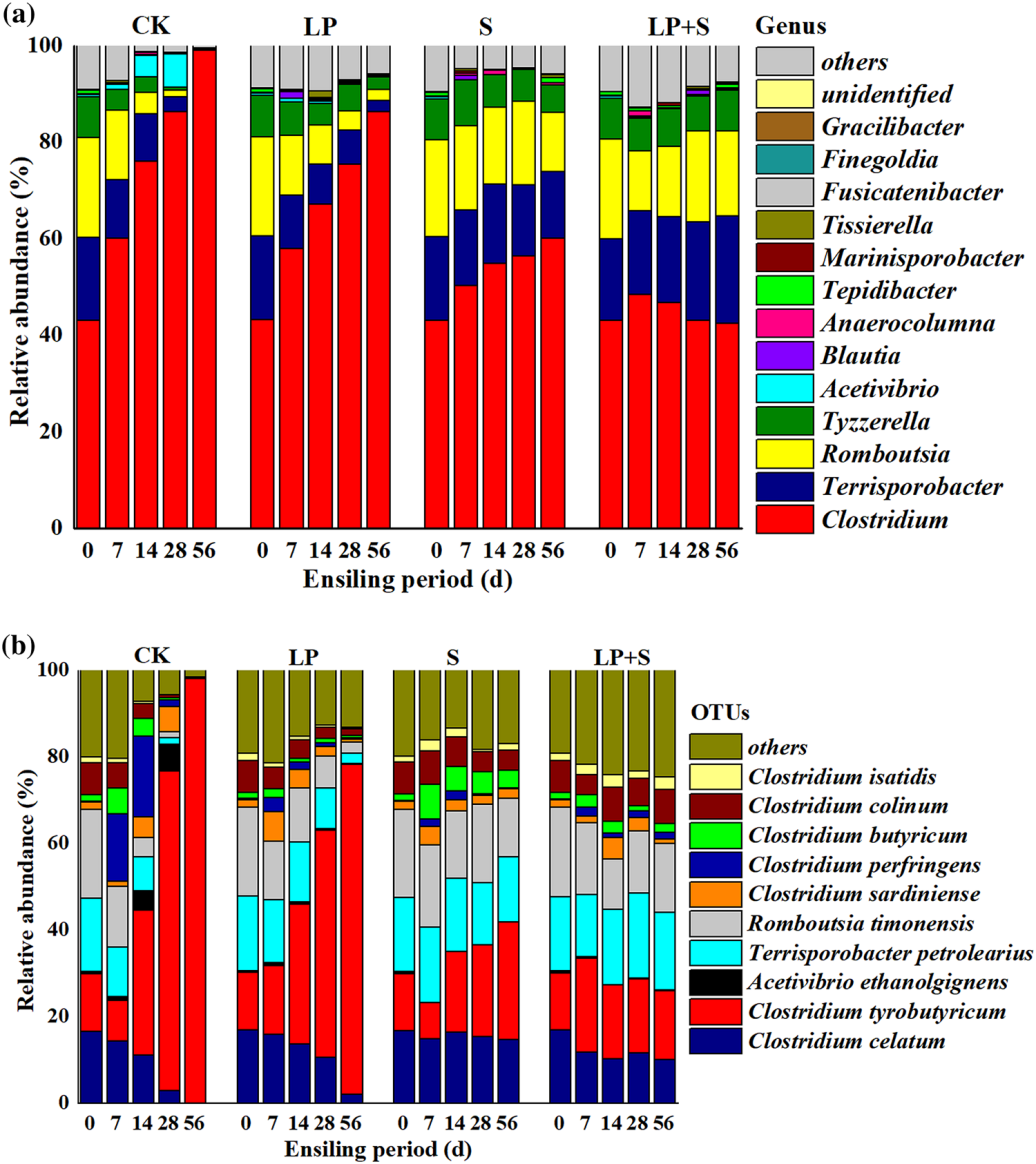
Relative abundance of the clostridial community at the genus (**a**) and species levels (**b**). CK, control; LP, *Lactobacillus plantarum*; S, sucrose; LP + S, the addition of both sucrose and *Lactobacillus plantarum*.

A new pair of specific primers (SJ-F and SJ-R) was applied to target the more diverse species within the class Clostridia. At the genus level, *Clostridium* (43.2%) was the most abundant genus identified in fresh alfalfa forage, followed by *Romboutsia* (20.7%) and *Terrisporobacter* (17.1%) (Fig. 5a). After ensiling, *Clostridium* still dominated the clostridial community of all silages, with its relative abundance continuously increasing over the ensiling period except for in LP + S from 14 to 56 days. Compared with S and LP + S, CK and LP showed higher relative abundances of *Clostridium* but lower relative abundances of *Terrisporobacter* and *Romboutsia* during ensiling. After 56 days of ensiling, the first three genera *Clostridium* (60.2% and 42.6%), *Terrisporobacter* (15.1% and 19.0%), and *Romboutsia* (9.6% and 18.7%) dominated the clostridial community in S and LP + S relative to *Clostridium* (99.0% and 86.3%) in CK and LP.

At the species level, *R. timonensis* (20.4%)*, T. pertrolearius* (17.0%), and *C. celatum* (16.7%) were prevalent species in fresh forage, whereas *C. butyricum* (1.55%) and *C. perfringens* (0. 25%) showed low relative abundances (Fig. 5b). After ensiling*, C. perfringens* rapidly increased, and *R. timonensis* and *T. pertrolearius* decreased in ensiled forage at 14 days in CK. *Clostridium butyricum* peaked at 7 days, and thereafter declined by 56 days in all silages and S showed a higher relative abundance of *C. butyricum* than CK during ensiling. After 7 days of ensiling, *C. tyrobutyricum* was the most abundant species in CK, and rapidly increased until the end of ensiling, whereas a slight decrease in *C. tyrobutyricum* was observed in LP + S during this period.

**Figure 6:**
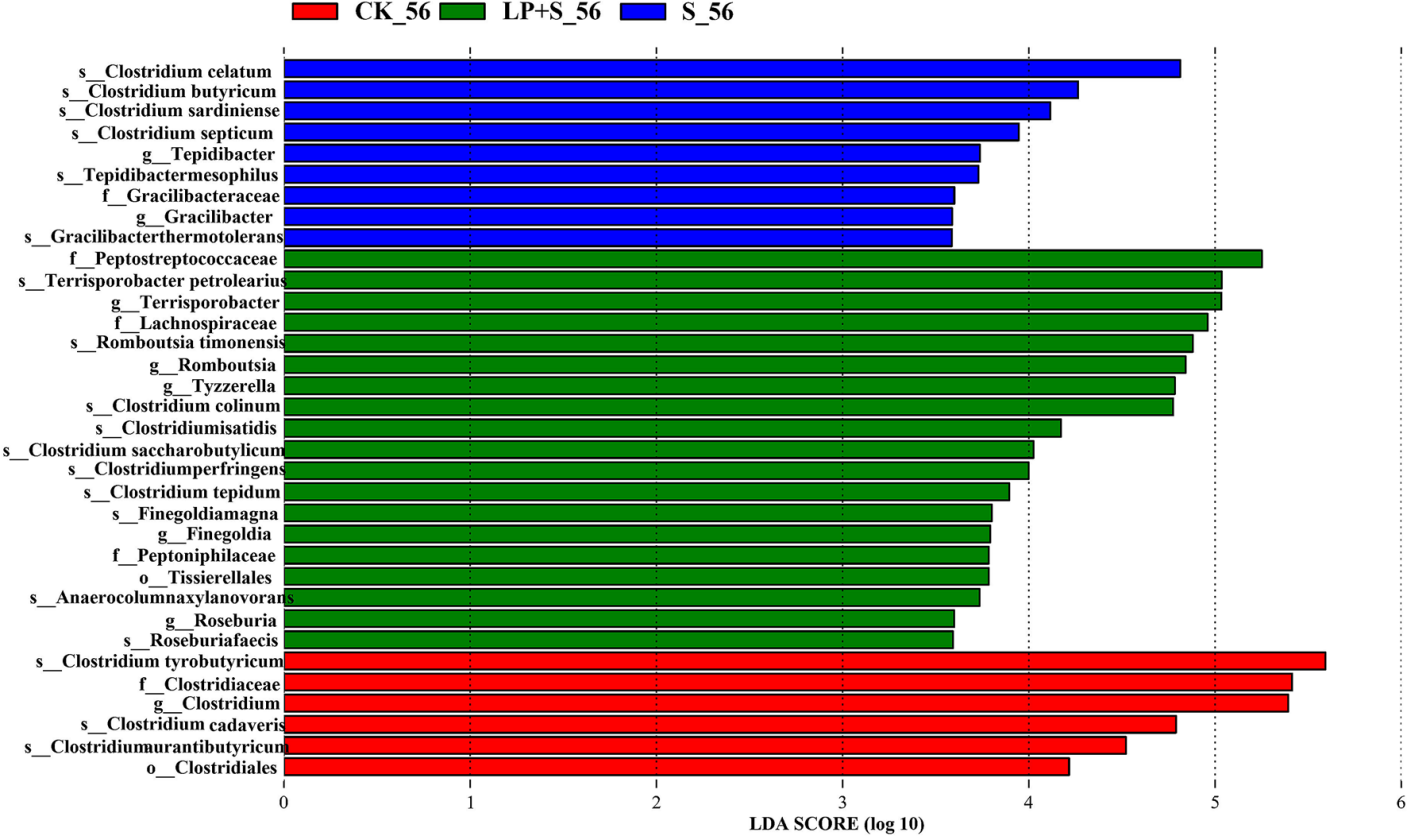
Comparison of clostridial variations using the LEfSe online tool for alfalfa silage at 56 days (CK, control; LP, *Lactobacillus plantarum*; S, sucrose; LP + S, the addition of both sucrose and *Lactobacillus plantarum*).

Differences in the clostridial community among groups were analyzed using the LEfSe method (Fig. 6). *Clostridium tyrobutyricum* and *Clostridium cadaveris* were higher in CK and *C. celatum* and *C. butyricum* were higher in S. *Terrisporobacter petrolearius* and *Romboutsia timonensis* were higher in LP + S.

### Correlation analysis between fermentation products and microbial community

**Figure 7:**
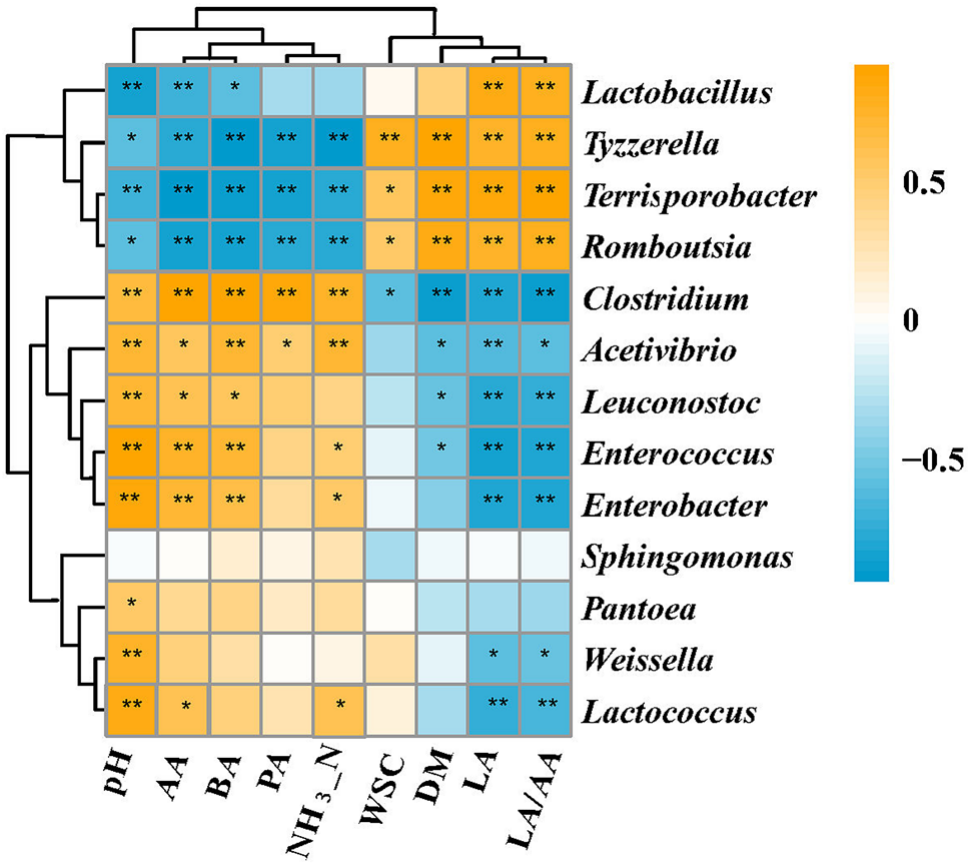
Spearman correlation analysis heatmap between the fermentation products and microbial community; **highly significant correlation at the 0.01 level; *highly significant correlation at the 0.05 level.

*Clostridium* (r = − 0.83), *Enterobacter* (r = − 0.84), *Lactococcus* (r = − 0.74) and *Enterococcus* (r = − 0.86) were negatively correlated with lactic acid content and positively correlated with pH with correlation coefficients of 0.68, 0.86, 0.85 and 0.92, respectively (Fig. 7). Acetic acid content was positively correlated with the genera *Clostridium* (r = 0.92), *Enterobacter* (r = 0.71) and *Enterococcus* (r = 0.75)*,* while was negatively correlated with the genera *Terrisporobacter* (r = − 0.96) and *Rombousia* (r = − 0.85)*.* Butyric acid content and NH_3_–N were positively correlated with the genera *Clostridium* (r = 0.90 and 0.78) and *Activibrio* (r = 0.73 and 0.74).

### Correlation analysis between the genera of single clostridia and other bacteria

**Figure 8:**
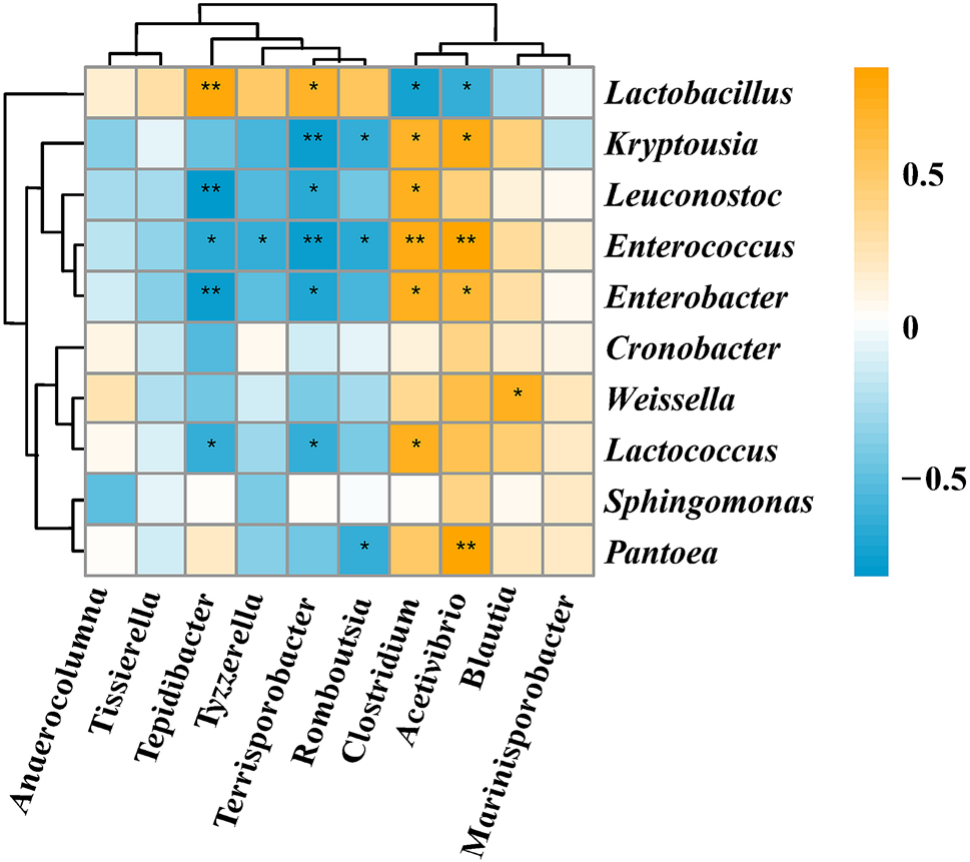
Spearman correlation analysis heatmap between the genera of single clostridia and other bacteria; **highly significant correlation at the 0.01 level; *highly significant correlation at the 0.05 level.

*Clostridium* was positively correlated with the genera *Enterobacter* (r = 0.60, pearson), *Enterococcus* (r = 0.63) and *Leuconostoc* (r = 0.59), while it was negatively correlated with *Lactobacillus* (r = − 0.57) (Fig. 8). *Terrisporobacter* was negatively correlated with the genera *Enterobacter, Enterococcus, Lactococcus and Leuconostoc,* with correlation coefficients of − 0.58, − 0.63, − 0.51 and − 0.55, respectively.

## Discussion

The process of lactic acid fermentation was changed to the typical clostridial fermentation in CK after 28 days of ensiling, as evidenced by the decrease in lactic acid content and increase in clostridial count and butyric acid content, resulting in a sudden rise in silage pH during this period. In contrast, fermentation parameters, such as silage pH, butyric acid, and NH_3_–N contents of ensiled alfalfa, indicated that clostridial fermentation was restrained to different extents, and high-quality silages were obtained in treated silages. These results agree with those of previous studies of alfalfa silages inoculated with LAB and sucrose^15,19,20^. These findings indicate that the extent of clostridial fermentation depends on the rate and extent of acidification after ensiling and fast acidification of silage was conducive to preventing clostridial fermentation.

LAB is regarded as the most crucial microorganisms in determining the final performance of silage. In this study, inoculation with *L. plantarum* directly resulted in its predominance in LP and LP + S, as reported previously^10,21^. *Lactobacillus plantarum* promoted the efficient utilization of sugars and quickly lowered the silage pH, thus ensuring the fermentation quality of ensiled alfalfa. The presence of enterobacteria in silage is undesirable because they ferment available sugars and amino acids to produce a mixture of acids (including acetic acid) and ammonia^1^. In this study, the higher *Enterobacter* abundance in CK may have been partly responsible for the higher acetic acid and NH_3_–N content in CK compared to in the treated silages. Although *Enterococcus* species such as *E. mundtii* and *E. gallinarum* increased rapidly and dominated the bacterial community in the CK throughout the ensiling period, they did not greatly affect the final quality of alfalfa silage, as evidenced by the high silage pH and low lactic acid content in CK during ensiling. Cai et al.^22^ reported that the inoculation of enterococci, including *E. mundtii*, did not improve the fermentation quality of ensiled alfalfa. It is well established that *Lactobacillus* will not become dominant until cocci-shaped LAB reach high numbers and produce large amounts of acids during the natural fermentation process^23^. In this study, sucrose additives enhanced the shift of dominant bacteria during ensiling from cocci to *Lactobacillus*, reflecting a decrease in the relative abundance of *E. mundtii* and *E. gallinarum* and increase in *L. plantarum*, *L. brevis* and *L. casei* in S compared to in CK, thus accelerating lactic acid fermentation and improving ensiling preservation.

Zheng et al.^20^ found that *Clostridium*, *Garciella* and *Rombousis* were the top three significant genera in alfalfa forage and dominated the ensiled alfalfa. In contrast, in this study, *Garciella* was not detected in epiphytic microflora or in ensiling microbes. The silage clostridia typically develop from spores that are almost located on the original forages because of contamination through soil or feces. However, this process is evidently certain to be quite variable, reflecting differences in environmental factors, such as crop species, climate, geographical location, and type of fertilizer applied^24^. Such discrepancies along with the silage condition may influence and change the clostridial community composition during the fermentation process. Generally, the proliferation of clostridia in silages is related to the ensiling conditions when initial lactic acid fermentation develops slowly or the extent of fermentation is unable to lower the silage pH efficiently^1^. In this study, all additives inhibited the proliferation of *Clostridium* to different extents and the abundance of this genus among all treatments at any time during ensiling was: CK > LP > S > LP + S. These differences may be caused by differences in the rate and extent of lactic acid fermentation and pH decrease from various treatments. In addition, compared with S and LP + S, the slower acidification and weaker lactic acid fermentation because of the sugar-deficient environment may have mainly accounted for the higher *Clostridium* abundance in CK and LP, particularly in CK.

Overall, *C. perfringens* and *C. butyricum* may be the main initiators of clostridial fermentation of alfalfa silage, as their relative abundance increased rapidly once the crop was ensiled for 7 days. As fermentation continued, *C. perfringens* increased and peaked at 14 days, whereas *C. butyricum* decreased. *Clostridium tyrobutyricum* quickly become the new dominant species after 7 days of ensiling. Zheng et al.^20^ observed that *C. perfringens*, *Garciella sp.*, and *Clostridium baratii* initiate clostridial fermentation at the early of ensiling process, whereas *C. tyrobutyricum* greatly contributes to clostridial fermentation of alfalfa silage in later stages. These results suggest that the difference in the epiphytic microflora of materials could affect and alter the initial fermentation succession of the clostridial community.

*Clostridium perfringens*, which is predominantly saccharolytic in character, can grow at relatively high pH (5.5 to 8.0)^25^. This may explain why *C. perfringens* plays a main role in the initial stage of clostridial fermentation in alfalfa silage. *Clostridium butyricum* proliferates at a somewhat lower pH range (> 4.5) than *C. perfringens* and typically ferments a wide range of carbohydrates^1^. In this study, *C. butyricum* peaked in all silages at 7 d and its relative abundance was highest in S (8.19%), followed by CK (6.07%) and LP + S (2.90%), indicating that supplementation of sufficient sugars stimulated the growth of *C. butyricum* in S to some extent. However, the resulting low pH caused by extensive acidification in LP + S inhibited the further growth of this species. The decrease in *C. butyricum* abundance in S from 14 to 56 days verified this hypothesis. *Clostridium tyrobutyricum*, because of its acid tolerance and ability to ferment lactic acid, has been shown to trigger clostridial fermentation of alfalfa silage in later stages of ensiling^20^. Indeed, *C. tyrobutyricum* developed intensively and dominated the clostridial community in CK from 14 to 56 days of fermentation, with a relative abundance of 33.5 to 98.0%, thus reducing the diversity of the clostridial community in CK during this period. The predominance of *C. tyrobutyricum* may also partly explain the decrease in lactic acid content in CK at 56 days. Although all additives inhibited the growth of *C. tyrobutyricum*, a weaker inhibitory effect was observed in LP compared to in S and LP + S, which was supported by the higher *C. tyrobutyricum* abundance in LP than in S and LP + S at 56 days. This suggests that *C. tyrobutyricum* triggered clostridial fermentation when the pH of the silage slowly decreased or when sugars necessary for acidification were insufficient. According to Thylin et al.^26^, both the rapid decline in pH and equally rapid original lactic acid fermentation were necessary to prevent the growth of *C. tyrobutyricum* in silage. In this study, the relative abundance of *C. tyrobutyricum* increased in S from 28 to 56 days of fermentation and decreased in LP + S from 7 to 14 days, although S and LP + S had similar pH ranges in this stage. These findings suggest that the higher lactic acid concentration in LP + S produced more undissociated acids with the low silage pH together achieving the complete inhibition of *C. tyrobutyricum*.

The two types of additives enhanced the fermentation process of ensiled alfalfa through different routes. Understanding the interactions between clostridia and other bacteria may contribute to the development of methods for controlling the fermentation process. *Clostridium* was negatively associated with *Lactobacillus*, but positively associated with the genera *Lactococcus*, *Enterococcus* and *Leuconostoc.* It is widely accepted that lactic acid-producing cocci (*Enterococcus, Lactococcus* and *Leuconostoc*) initiate lactic acid fermentation at early stage of ensiling^21^. Additionally, clostridia theoretically begin to proliferate by utilizing a variety of organic compounds including carbohydrates, proteins and lactic acid, which cannot be stopped unless an anaerobically stable pH is reached^1^. In this case, metabolites such as ammonia and volatile fatty acids, may counteract the part acidification and prevent the rapid decrease in pH, which seems to concurrently create the suitable conditions for the growth of acid-intolerant LAB (*Enterococcus, Lactococcus* and *Leuconostoc*). However, with the accumulation of lactic acid and reduction of silage pH, *Lactobacillus* outcompete other bacteria and begin to dominate ensiling fermentation, with concomitant decreases in *Enterococcus, Lactococcus*, *Leuconostoc*, and *Clostridium* abundance.

## Conclusions

Untreated silages underwent severe clostridial fermentation, whereas all additives, particularly LP + S, decreased the silage pH and restrained clostridial fermentation. *Clostridium perfringens* and *C. butyricum* may be the main initiators of clostridial fermentation, after which *C. tyrobutyricum* acts as a promoter of fermentations until the end of ensiling. Slow acidification promoted the vigorous growth of *C. tyrobutyricum* of silage in the later stage, which was mainly responsible for clostridial fermentation of alfalfa silage.

## Materials and methods

### Plant material and silage making

Alfalfa (*Medicago sativa* L., WL363HQ) was planted at the Zhuozhou experimental station (39°28′N, 115°510′E) of China Agricultural University and treated with no fertilizers and herbicides during growth. The organic matter in the experimental plots was approximately 13.5 g/kg; effective N, P and K in the soil were 81.2, 24.1 and 125.5 mg/kg respectively. Fourth-cut alfalfa was harvested at squaring stage from the three test plots of similar growth and chopped into about 1 to 2 cm length by a hand chopper. Sucrose (analytical reagent; Beijing Chemical Industries Ltd, Beijing, China) and *Lactobacillus plantarum* strain L12FL5 (NCBI accession number: KM005154) were used as additives for ensiling preparation. The LP strain isolated from alfalfa silage could grow well under low pH conditions and possess high acidification activity. The chopped alfalfa was mixed and separated into equal part for four treatments comprising no additives (CK); application of sucrose (S); application of *Lactobacillus plantarum* (LP); combination of LP and S (LP + S). The LAB strain was dissolved in sterile distilled water to an equivalent of 10^5^ cfu/g FM and an equal volume of distilled water was applied to dissolve S at 20.0 g/kg FM. Silage sprayed with the same amount of distilled water served as a control. Each sample (350 g) was packed manually into vacuum plastic bags, sealed quickly using a vacuum package machine (BH 950, Matsushita, Tokyo, Japan), and then stored at ambient temperature (15–25 °C). Triplicate silos for each treatment were opened after 7, 14, 28 and 56 days of ensiling.

### Analysis of fermentation quality, chemical and microbial composition

For the nutritional composition and fermentation parameters, a wet sample (20 g) randomly collected from each silo was thoroughly blended with 180 mL of sterilized distilled water and then filtered through 4 layers of cheesecloth and a qualitative filter paper. The filtrates were stored at − 80 °C for further analysis. The pH was determined using a glass electrode pH meter (Mettler Toledo S20, Switzerland). NH_3_–N was analyzed by the method of Broderick & Kang^27^. The concentrations of organic acid including lactic, acetic, propionic, and butyric acids were measured by high performance liquid chromatography (LC-10A, Shimadzu, Japan). The parameters were as follows: column, Shodex RS PAK KC-811S-DVB; mobile phase, 3 mmol/L HClO_4_; flow rate, 1.0 mL/min; oven temperature, 50 °C. The dry matter and crude protein were calculated following the procedure of AOAC^28^. WSC was measured as described by the method of Mcdonald and Henderson^29^. For the microbial abundance, a wet sample (20 g) randomly collected from each silo was serially diluted using 180 mL sterilized distilled water and serially diluted from 10^–1^ to 10^–7^ in sterilized water before microbial enumeration. LAB counts were measured by plate count on de Man, Rogosa, and Sharpe agar (Difco Laboratories, Detroit, MI, USA) after incubation at 37 °C for 48 h under anaerobic conditions (TE-HER Hard Anaerobox, ANX-1; Hirosawa Ltd, Tokyo, Japan). Enterobacteria were counted on Blue Light Broth agar (Nissui Ltd., Tokyo, Japan) after incubation at 37 °C for 48 h. Clostridia were analyzed on reinforced clostridia agar (Aobox, Beijing, China) using the Hungate technique^30^. Cold-water extracts were heated to 80 °C for 10 min to inactivate the vegetative cells and to trigger the germination of spores, and 500 μL aliquots of the diluted extracts were inoculated into Hungate tubes. The tubes were rolled on ice and incubated at 37 °C for 5 days. Black colonies were identified as Clostridia.

### DNA extraction

Each sample (20 g) were combined with 80 mL of sterile 0.85% NaCl solution for 2 h at 4 °C, then was filtered through two layers cheesecloth and centrifuged at 12,000 × *g* at 4 °C for 15 min. The supernatant was discarded and the microbial pellets were stored at − 80 °C until DNA extraction. Triplicate samples of each treatments were measured individually. Total DNA were extracted using PowerSoil DNA Isolation Kit (MoBio Laboratories, Carlsbad, CA) following the manufacturer’s guideline.

### Sequencing

The universal primer pair 338F (5′-GTACTCC-TACGGGAGGCAGCA-3′) and 806R (5′-GTGGACTACHVGGGTWT-CTAAT-3′) were adopted to target the V3–V4 regions of the bacterial 16S rRNA gene. The specific primer pair SJ-F (5′-CGGTGAAATGCG-TAGAKATTA-3′) and SJ-R (5′-CGA-ATTAAACCACATGCTCCG-3′) were adopted to target the half V4 and total V5 regions of clostridial 16S rRNA gene^14^. Both primer pairs had 12-bp barcodes unique to each sample, in order to enable the pooling of all PCR products for sequencing and the subsequent assignation of sequence reads to their respective samples^31^. The PCR program was 94 °C for 2 min, 30 cycles of 94 °C for 30 s, 57 °C for 30 s, and 72 °C for 30 s, with a final extension of 72 °C for 10 min. PCR products were extracted from 2% agarose gels, and then purified using the AxyPrep DNA Gel Extraction Kit (Axygen Biosciences, Union City, CA, USA) and quantified using QuantiFluor-ST (Promega, Madison, WI, USA) according to the manufacturer’s instructions. The PCR products were pooled in equimolar ratios and paired-end sequenced with an Illumina Miseq PE300 platform (Illumina, San Diego, CA, USA).

### Bioinformatic analysis

To obtain high-quality reads, sequences shorter than 200 base pairs and those with low-quality scores (≤ 20) for barcodes and primers were discarded according to the Quantitative Insights Into Microbial Ecology standard (version 1.7.0, https://qiime.org/index.html) as described previously^32^. DNA sequences were grouped into OTUs defined as sequences with > 97% sequence identity using the UPRASE software package (version7.0.1001, https://drive5.com/uparse/). Taxonomic classification of each OTU representative sequence was determined using the Ribosomal Database Project Classifier (version 2.2,) using a minimum bootstrap threshold of 80%. Alpha diversity in https://sourceforge.net/projects/rdp-classifier/dices including Shannon index, Chao1 richness estimator and the Good’s coverage) were calculated using MOTHUR software (version1.30.1, https://www.mothur.org/wiki/Classify.seqs)^33^. Principal component analysis was performed by the permutational multivariate analysis of variance based on Euclidean distance. The graph presentations were generated using the R program (Version 3.6.0, https://www.r-project.org). To explore relationships between the microbial community and fermentation products as well as the genera of single clostridia and other bacteria, Spearman’s rank correlation matrix was generated by calculating the Spearman’s correlation coefficient. The level of high significance was set to *P*-value of less than 0.05 with |r|> 0.40. The correlation matrix was visualized as a heatmap produced at the genus level using the R program (version 3.6.0, https://www.r-project.org) vegan package. The LEfSe analysis (Galaxy Version 1.0, https://huttenhower.sph.harvard.edu/galaxy/) was conducted to determine the differentially abundant taxonomies among different treatments by coupling one-way analysis of variance with nonparametric factorial Wilcoxon sum-rank test for statistical significance using python (version 2.7, https://www.python.org). Linear discriminant analysis (LDA) scores more than 2.0 were speculated to have different abundance.

### Statistical analysis

All microbial counts were log-transformed based on a fresh weight before statistical analysis. Data on fermentation characteristics, microbial counts, and alpha diversity of bacterial and clostridial community in alfalfa silage were evaluated by two-way ANOVA with the main effects of additives, storage period and additives by storage period interaction using the general linear model procedure of spss20.0. Turkey’s multiple range method was adopted to compare the difference at 0.05 significant level.

## References

[1] Pahlow G, Muck RE, Driehuis F, Elferink SJO, Spoelstra SF (2003). Microbiology of ensiling. Silage Sci. Technol..

[2] Gibson T (1965). Clostridia in silage. J. Appl. Bacteriol..

[3] Zheng M (2018). The effect of cultivar, wilting and storage period on fermentation and the clostridial community of alfalfa silage. Ital. J. Anim. Sci..

[4] Cremonesi P, Vanoni L, Silvetti T, Morandi S, Brasca M (2012). Identification of Clostridium beijerinckii, Cl. butyricum, Cl. sporogenes, Cl. tyrobutyricum isolated from silage, raw milk and hard cheese by a multiplex PCR assay. J. Dairy Res..

[5] Myllykoski J (2009). Type C bovine botulism outbreak due to carcass contaminated non-acidified silage. Epidemiol. Infect..

[6] Guo X (2018). Profiling of metabolome and bacterial community dynamics in ensiled Medicago sativa inoculated without or with Lactobacillus plantarum or Lactobacillus buchneri. Sci. Rep..

[7] Bryant MP, Burkey LA (1956). The characteristics of lactate-fermenting sporeforming anaerobes from silage. J. Bacteriol..

[8] Flythe MD, Russell JB (2004). The effect of pH and a bacteriocin (bovicin HC5) on Clostridium sporogenes MD1, a bacterium that has the ability to degrade amino acids in ensiled plant materials. FEMS Microbiol. Ecol..

[9] Rossi F, Dellaglio F (2007). Quality of silages from Italian farms as attested by number and identity of microbial indicators. J. Appl. Microbiol..

[10] Zheng M (2018). The effect of cultivar, wilting and storage period on fermentation and the clostridial community of alfalfa silage. Ital. J. Anim. Sci..

[11] Hamm AC (2016). Bacterial communities of an agricultural soil amended with solid pig and dairy manures, and urea fertilizer. Appl. Soil Ecol..

[12] Yan Y (2019). Microbial community and fermentation characteristic of Italian ryegrass silage prepared with corn stover and lactic acid bacteria. Biores. Technol..

[13] Yuan X, Li J, Dong Z, Shao T (2020). The reconstitution mechanism of napier grass microiota during the ensiling of alfalfa and their contributions to fermentation quality of silage. Biores. Technol..

[14] Hu XL, Wang HY, Wu Q, Xu Y (2014). Development, validation and application of specific primers for analyzing the clostridial diversity in dark fermentation pit mud by PCR-DGGE. Biores. Technol..

[15] Liu Q, Dong Z, Shao T (2018). Effect of additives on fatty acid profile of high moisture alfalfa silage during ensiling and after exposure to air. Anim. Feed Sci. Technol..

[16] Zhang Q, Yu Z, Wang X (2015). Isolating and evaluating lactic acid bacteria strains with or without sucrose for effectiveness of silage fermentation. Grassl. Sci..

[17] Dong M, Li Q, Xu F, Wang S, Li W (2020). Effects of microbial inoculants on the fermentation characteristics and microbial communities of sweet sorghum bagasse silage. Sci. Rep..

[18] Wang Y (2019). The bacterial community and fermentation quality of mulberry (Morus alba) leaf silage with or without Lactobacillus casei and sucrose. Biores. Technol..

[19] Zhang Q, Yang HJ, Yu Z (2017). Effects of sucrose, formic acid and lactic acid bacteria inoculant on quality, in vitro rumen digestibility and fermentability of drooping wild ryegrass (Elymus nutans Griseb.) silage. J. Anim. Feed Sci..

[20] Zheng ML, Niu DZ, Jiang D, Zuo SS, Xu CC (2017). Dynamics of microbial community during ensiling direct-cut alfalfa with and without LAB inoculant and sugar. J. Appl. Microbiol..

[21] Yang L, Yuan X, Li J, Dong Z, Shao T (2019). Dynamics of microbial community and fermentation quality during ensiling of sterile and nonsterile alfalfa with or without Lactobacillus plantarum inoculant. Biores. Technol..

[22] Cai Y (1999). Identification and characterization of Enterococcus species isolated from forage crops and their influence on silage fermentation. J. Dairy Sci..

[23] Langston C, Bouma C (1960). A study of the microorganisms from grass silage: II. The lactobacilli. Appl. Microbiol..

[24] Gibson T (1965). Clostridia in silage. J. Appl. Microbiol..

[25] Jia Zhen LY, Hwang C-A, Huang L (2020). Effect of combination of Oxyrase and sodium thioglycolate on growth of Clostridium perfringens from spores under aerobic incubation. J. Food Microbiol..

[26] Thylin I, Schuisky P, Lindgren S, Gottschal JC (1995). Influence of pH and lactic acid concentration on Clostridium tyrobutyricum during continuous growth in a pH-auxostat. J. Appl. Microbiol..

[27] Broderick GA, Kang JH (1980). Automated simultaneous determination of ammonia and total amino acids in ruminal fluid and in vitro media. J. Dairy Sci..

[28] Chemists, A. o. O. A. Official methods of analysis. (1990).

[29] Mcdonald P, Henderson AR (2010). Determination of water-soluble carbohydrates in grass. J. Sci. Food Agric..

[30] Hungate & R., E. Chapter IV a Roll Tube Method for Cultivation of Strict Anaerobes. Methods in Microbiology **3,** 117–132 (1969).

[31] Neher DA, Weicht TR, Bates ST, Leff JW, Fierer N (2013). Changes in bacterial and fungal communities across compost recipes, preparation methods, and composting times. PLoS ONE.

[32] Bokulich NA (2013). Quality-filtering vastly improves diversity estimates from Illumina amplicon sequencing. Nat. Methods.

[33] Schloss PD (2009). Introducing mothur: open-source, platform-independent, community-supported software for describing and comparing microbial communities. Appl. Environ. Microbiol..

